# The BenBioDen database, a global database for meio-, macro- and megabenthic biomass and densities

**DOI:** 10.1038/s41597-020-0551-2

**Published:** 2020-06-29

**Authors:** Tanja Stratmann, Dick van Oevelen, Pedro Martínez Arbizu, Chih-Lin Wei, Jian-Xiang Liao, Mathieu Cusson, Ricardo A. Scrosati, Philippe Archambault, Paul V. R. Snelgrove, Patricia A. Ramey-Balci, Brenda J. Burd, Ellen Kenchington, Kent Gilkinson, Rénald Belley, Karline Soetaert

**Affiliations:** 1 NIOZ Royal Netherlands Institute for Sea Research, Department of Estuarine and Delta Systems, and Utrecht University, P.O. Box 140, 4400 AC, Yerseke, The Netherlands; 2 Utrecht University, Department of Earth Sciences, Vening Meineszgebouw A, Princetonlaan 8a, 3584 CB, Utrecht, The Netherlands; 3 HGF MPG Joint Research Group for Deep-Sea Ecology and Technology, Max Planck Institute for Marine Microbiology, Celsiusstr. 1, 28359 Bremen, Germany; 4 German Centre for Marine Biodiversity, Senckenberg am Meer, Südstrand 44, 26382 Wilhelmshaven, Germany; 5 Institute of Oceanography, National Taiwan University, No. 1, Sec. 4, Roosevelt Road, Taipei, 105 Taiwan; 6 Département des sciences fondamentales et Québec-Océan, Université du Québec à Chicoutimi, Boulevard de l’Université, Chicoutimi, QC G7H 2B1 Canada; 7 Department of Biology, St. Francis Xavier University, 2320 Notre Dame Ave., Antigonish, NS B2G 2W5 Canada; 8 ArcticNet & Québec-Océan/Takuvik, Université Laval, pavillon Alexandre-Vachon 1045, av. de la Médecine, Québec, QC G1V 0A6 Canada; 9 Department of Ocean Sciences and Biology, Memorial University of Newfoundland, Marine Lab Rd., St. John’s, NL A1C 5S7 Canada; 10 College of Sciences, Koç University, Rumelifeneri Yolu 34450, Sarıyer, Istanbul, Turkey; 11 Institute of Ocean Sciences, Fisheries and Ocean Canada, P.O. Box 6000, Sidney, BC V8L 5T5 Canada; 12 Bedford Institute of Oceanography, Fisheries and Ocean Canada, P.O. Box 1006, 1 Challenger Dr., Dartmouth, NS B2Y 4A2 Canada; 13 Northwest Atlantic Fisheries Centre, Fisheries and Ocean Canada, 80 East White Hills, St. John’s, NL A1C 5 × 1 Canada; 14 Maurice Lamontagne Institute, Fisheries and Oceans Canada, 850 Route de la Mer, Mont-Joli, QC G5H 3Z4 Canada

**Keywords:** Ecosystem services, Marine biology

## Abstract

Benthic fauna refers to all fauna that live in or on the seafloor, which researchers typically divide into size classes meiobenthos (32/64 µm–0.5/1 mm), macrobenthos (250 µm–1 cm), and megabenthos (>1 cm). Benthic fauna play important roles in bioturbation activity, mineralization of organic matter, and in marine food webs. Evaluating their role in these ecosystem functions requires knowledge of their global distribution and biomass. We therefore established the BenBioDen database, the largest open-access database for marine benthic biomass and density data compiled so far. In total, it includes 11,792 georeferenced benthic biomass and 51,559 benthic density records from 384 and 600 studies, respectively. We selected all references following the procedure for systematic reviews and meta-analyses, and report biomass records as grams of wet mass, dry mass, or ash-free dry mass, or carbon per m^2^ and as abundance records as individuals per m^2^. This database provides a point of reference for future studies on the distribution and biomass of benthic fauna.

## Abstract (summary)

**Table:** 

Measurement(s)	marine benthic feature • organic material • mass density
Technology Type(s)	digital curation
Factor Type(s)	geographic location • water depth • organism size class
Sample Characteristic - Organism	meiobenthos • macrobenthos • megabenthos
Sample Characteristic - Environment	marine benthic biome • ocean
Sample Characteristic - Location	Atlantic Ocean • Pacific Ocean • Arctic Ocean • Southern Ocean • Indian Ocean

Machine-accessible metadata file describing the reported data: 10.6084/m9.figshare.12481985

## Background & Summary

Benthic fauna, the fauna living in (infauna) or on the seafloor (epifauna), includes size classes known as metazoan meiobenthos, metazoan macrobenthos, and megabenthos. Metazoan meiobenthos passes through a 500 µm or 1 mm^1^ mesh and is retained on a sieve with a mesh size of 32 µm (deep-sea meiobenthos) or 63 µm (shallower water depth). Frequently, however, no upper sieve is used. Meiobenthos can actively rework sediment particles and build microscale burrows in the sediment^2^. Additionally, it represents a food source for juvenile and small adult fish^3,4^. Meiobenthos also contributes to organic matter mineralization and nutrient regeneration by stimulating the microbial community^5^. Metazoan macrobenthos passes through a 1 cm mesh and is retained on sieves with a mesh size of 250 or 300 or 500 µm (depending on the study). Macrobenthos is an important bioturbator that reworks the sediment (bioturbation *sensu*^6^), and in doing so alters the texture of the sediment^7^ and reduces slope failure^8^. It can be ecosystem engineers, i.e., organisms that alter the physical environment to change directly or indirectly the availability of resources to other organisms^9^, and modify hydrodynamics. Megabenthos or fauna larger than 1 cm includes organisms such as scleractinian corals or sponges that form biological structures and thus provide new habitats for associated fauna^10,11^. Other examples of megabenthos assemblages are oyster reefs and mussel beds that create biogeochemical hotspots for the burial of organic matter and the recycling of nutrients^12–14^. Additionally, mussels, cockles, oysters, but also sea cucumbers, are part of the human diet.

Despite their ecological importance, benthic ecosystems face increasing pressures from fishing, pollution and litter disposal, gas and oil exploration, extraction of minerals, development of coastlines, shipping, tourism, invasive species, and wind farms^15–17^. Sea-level rise can force intertidal habitats, such as salt marshes and tidal flats, to migrate landwards where they may be squeezed against artificial coastal structures^18^. This “coastal squeeze” leads to the loss of intertidal habitats and macrobenthic biomass^19,20^. Furthermore, ocean acidification will strongly impact tropical and cold-water coral reefs^21–23^ and calcareous fauna such as bivalves, gastropods, bryozoans, echinoderms, and foraminifera^24^. A combination of changes in pH, temperature, and oxygenation will even affect the export flux of particulate organic carbon (POC) to the seafloor^25^ and subsequently result in decreased benthic biomass^26^.

Evaluation of the severity of these threats and climate change for the benthic ecosystem on a global scale requires quantifying the role of benthos and its biomass and density in particular. Here, we introduce the open access “BenBioDen database”^27^ that, in comparison to previous databases by, e.g., Rex *et al*.^28^ and Wei *et al*.^29^, makes the benthic biomass and abundance records freely available and describes the data selection procedure transparently. Furthermore, this database includes records from the whole globe and not only from specific geographic regions, like the *MarLIN – The Marine Life Information Network* database (https://www.marlin.ac.uk/) or the *OSPAR Data & Information Management System* (ODIMS) database (https://odims.ospar.org/).

The “BenBioDen database” reports 1,445 benthic biomass and 2,085 benthic density studies and datasets identified following standardized procedures for systematic reviews and meta-analyses^30^. As a result, we extracted 11,792 georeferenced records of benthic biomass (1,240 metazoan meiobenthos records, 9,292 macrobenthos records, and 1,260 invertebrate megabenthos records) and 51,559 georeferenced records of benthic densities (4,129 metazoan meiobenthos records, 46,389 macrobenthos records, and 1,041 invertebrate megabenthos records) from 384 and 600 selected studies, respectively. We report benthic biomass as g wet mass (WM) m^−2^, as g dry mass (DM) m^−2^, as g ash-free dry mass (AFDM) m^−2^ or as g carbon (C) m^−2^. All biomass and density data records include further information about the mesh size used to separate meiobenthos from macrobenthos and megabenthos, and macrobenthos from megabenthos, and the sampling gear. In this way, researchers can decide whether they wish to exclude specific studies that do not match organism size criteria or sampling gear criteria. The database provides an important point of reference for future studies on the distribution and biomass of benthos and may also stimulate future sampling campaigns by indicating undersampled locations and water depth.

## Methods

**Fig. 1:**
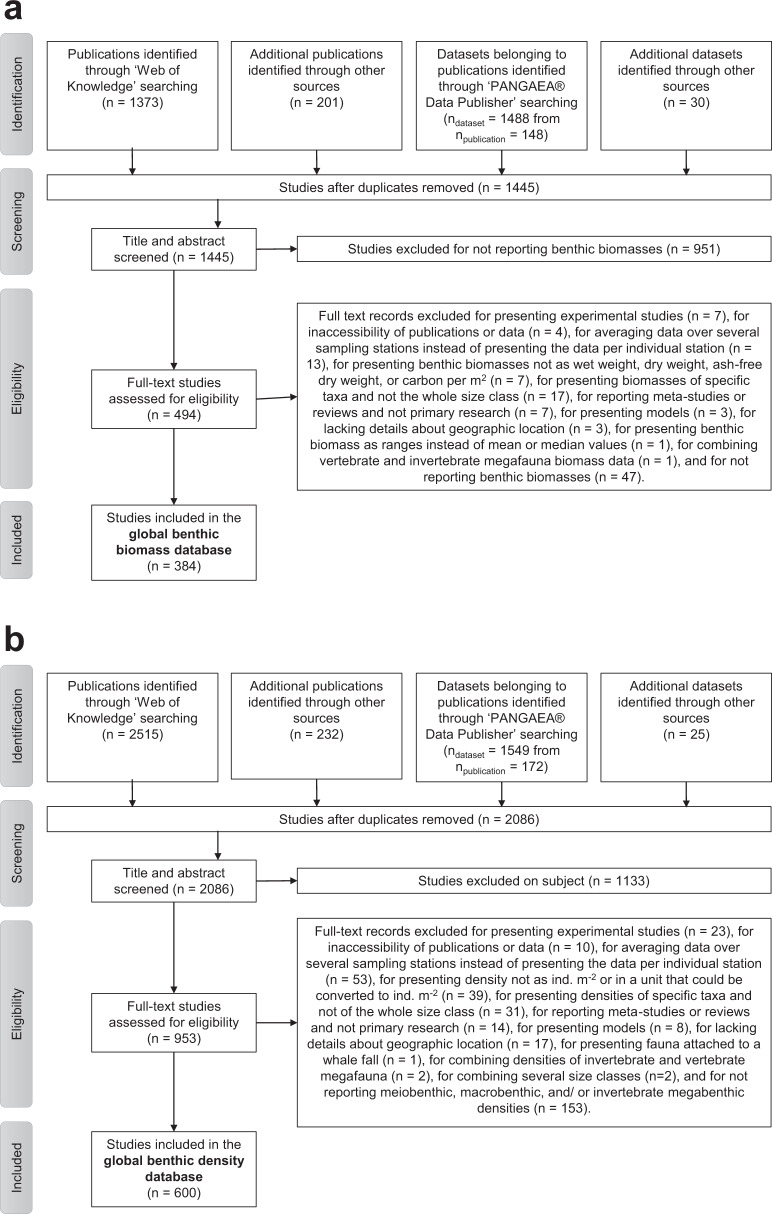
Flow chart explaining how the database was compiled. It shows how publications and datasets were identified and which selection criteria were used to exclude studies from the final “BenBio” part of the BenBioDen database (panel a) and the final “BenDen” part of the BenBioDen database (panel b).

In April and May 2019, we compiled the “BenBio” part of the “BenBioDen database” following the “Preferred Reporting Items for Systematic reviews and Meta-Analyses” (PRISMA) Statement for systematic reviews and meta-analyses^30^ (Fig. 1a). In the first PRISMA step, the “Identification” step, we identified 1,373 articles in the *Web of Science* using the key words “marine meiofauna biomass”, “marine macrofauna biomass”, “marine megafauna biomass”, “marine meiobenth* biomass”, “marine macrobenth* biomass”, “marine megabenth* biomass”, “nematode biomass”, and “benthic ‘standing stock’”. We located an additional 201 publications based on expert knowledge. A search of the *PANGAEA*^(R)^ *Data Publisher* (https://www.pangaea.de/) identified 1,488 datasets representing 148 publications using the key words “meiofauna biomass”, “macrofauna biomass” and “megafauna biomass”. Further 30 datasets were found in the *EOL data archive* (http://data.eol.ucar.edu/), through citations in review papers, and based on expert knowledge. After removing duplicates, we screened the titles and abstracts of 1,445 studies (Online-only Table 1) in PRISMA step 2 (“Screening”; Fig. 1a). This step excluded 951 studies because they did not report biomass values. In the Eligibility step (step 3; Fig. 1a), we assessed full texts of 494 studies for eligibility and excluded 110 studies because they did not report biomass, the publications or data were not accessible, or they did not report benthic biomass in appropriate units (g WW m^−2^, g DW m^−2^, g AFDW m^−2^, g or mol C m^−2^). Further reasons for excluding full texts included combining benthic biomass for several size classes, reporting benthic biomass for particular taxa rather than the whole size class, presenting biomass for faunal assemblages and/or a group of sampling stations rather than for individual stations, not presenting primary research or lacking geographical details about sampling stations. We also excluded studies that estimated benthic biomass using modelling approaches, that conducted manipulative experiments, or did not report benthic biomass as single values, means or median values, but instead as ranges. The final “BenBio” part included 384 studies from which we extracted 11,792 georeferenced benthic biomass entries (Online-only Table 1; Fig. 1a).

The Benthos Density, i.e. “BenDen”, part of the “BenBioDen” database was established in July and August 2019 following the PRISMA Statement for systematic reviews and meta-analyses^30^ (Fig. 1b). In the Identification step, we found 2,515 articles in the *Web of Science* using the key words “meiofauna abundance”, “meiobenthos abundance”, “macrofauna abundance”, “macrobenthos abundance”, “megafauna abundance”, “megabenthos abundance”, “meiofauna Arctic Ocean”, “meiofauna Atlantic Ocean”, “meiofauna Black Sea”, “meiofauna Gulf of Mexico”, “meiofauna Indian Ocean”, “meiofauna Mediterranean Sea”, “meiofauna Pacific Ocean”, “meiofauna Southern Ocean”, “meiofauna Red Sea”, “meiofauna Pacific Ocean”, “megafauna Southern Ocean”, “megafauna Red Sea”, “megafauna Pacific Ocean”, “megafauna Mediterranean Sea”, “megafauna Indian Ocean”, “megafauna Black Sea”, “megafauna Gulf of Mexico”, “megafauna Atlantic Ocean”, “megafauna Arctic Ocean”, “macrofauna Arctic Ocean”, “macrofauna Atlantic Ocean”, “macrofauna Black Sea”, “macrofauna Southern Ocean”, “macrofauna Red Sea”, “macrofauna Pacific Ocean”, “macrofauna Gulf of Mexico”, “macrofauna Indian Ocean”, and “macrofauna Mediterranean Sea”. Expert knowledge identified a further 232 publications. Consulting *PANGAEA*^(R)^ *Data Publisher* (https://www.pangaea.de/) identified 1,549 datasets from 172 publications using the key words “meiofauna abundance”, “macrofauna abundance” and “megafauna abundance”. Expert knowledge or unpublished datasets added a further 21 datasets. After removal of duplicates, the “Screening” step filtered 2,086 titles and abstracts (Online-only Table 1; Fig. 1b) and excluded 1,133 studies because they did not report benthic densities. The third PRISMA step (“Eligibility”; Fig. 1b) assessed 953 studies and excluded 353 studies because they did not report metazoan meiobenthic, macrobenthic, or invertebrate megabenthic densities or they combined multiple size classes or sampling stations. We excluded other studies in the database that reported experimental studies, were inaccessible, or reported densities in a unit other than ind. m^−2^ or a unit that could be converted to ind. m^−2^, or reported densities for specific taxa instead of the entire size class. Studies were also excluded when they reported meta-studies or reviews rather than primary research, presented results of models, lacked sufficient geographical detail about sampling locations, or reported fauna associated with whale falls. The final “BenDen” part consisted of 600 studies from which we extracted 51,559 georeferenced benthic density records (Online-only Table 1; Fig. 1b).

For 12% (BioBen part) and 4% (BioDen part) of all data records, no exact sampling location in geographical coordinates (latitude, longitude) was indicated. For these cases, we approximated the coordinates of the sampling locations using Google Maps based on information about sampling area or based on maps presented in the original publications. We labelled these data records as ‘approximated location’.

For studies that presented biomasses in several units, such as WM *and* DM, we report the data only once (preferred units: WM > DM > AFDM > C). The authors of this study intended to report all data records in the ‘raw’ units in which benthic fauna was measured initially. Whenever unknown conversion factors precluded calculating biomass back to ‘raw’ units, we noted this issue in the database using the label ‘converted data’ and listed references for the individual biomass conversion factors in the database. Furthermore, we prepared Table 1 that reports all literature used by the authors of the original studies to convert their biomasses size-class dependent to WM, DM, AFDM, and C content.

**Table 1:** References of biomass conversion factors to calculate metazoan meiobenthic, macrobenthic, and megabenthic biomasses as wet mass (WM), dry mass (DM), ash-free dry mass (AFDM), and C content (C).

Size class	References for biomass conversion to			
	WM	DM	AFDM	C
Meiobenthos	^38–42^	^38–40,42–58^	^38,39,59^	^1,38–42,57,59–79^
Macrobenthos	^41,80^	^38,39,53,62,81–88^	^83,89–93^	^38–41,60,65,67,68,80,81,91–106^
Megabenthos				^81,91,100,103,104,107–109^

The authors of the various studies compiled in this database sometimes used different lower and upper limits (in mm) for mesh sizes of nets and/or sieves to define the size class. Whenever an original study reported a lower and/or upper limit mesh size, we included this information in the database as ‘sieve mesh size (mm) lower limit’ and ‘sieve mesh size (mm) upper limit’. Studies lacking this information were scored as NA.

For those studies that reported data as mean or median ± error terms, we incorporated only mean or median values into the database. In all cases that did not report benthic biomasses and/or densities in the text or in tables, but presented them in figures, we extracted biomass and/or density values from these figures using ImageJ^31^.

## Data Records

The BenBioDen database is openly accessible in the *Dryad Digital Repository*^27^ and includes two txt.files, i.e. the *List of studies for BenBio database* file and the *List of studies for BenDen database* file, and two csv.files, i.e., the *BenBio database* file and the *BioDen database* file. The *List of studies* files list all 3,531 studies alphabetically (benthic biomasses: 1,445 studies; benthic densities: 2,086 studies) which we identified in the “Identification” step of the systematic review after removing all duplicates. Each data entry in the BioBenDen database contains information about the region where the biomasses and/or densities were sampled and the corresponding ocean, the geographical location (latitude, longitude), whether geographic location was exactly known or approximated, water depth (in m), and a depth range following Dunne *et al*.^32^. Dunne and co-authors divided the ocean in near-shore areas that stretch to 50 m water depth, continental shelves from > 50 to 200 m water depth, continental slopes from > 200 to 2,000 m water depth, and continental rises/abyssal plains > 2,000 m water depth. The database indicates whether we determined the biomasses as WM, DM, AFDM, or C content; densities are reported as ind. m^−2^. The database also reports the specific size class (metazoan meiobenthos, macrobenthos, invertebrate megabenthos), the mesh size of the sieves used by the authors of the studies to separate the different size classes and the sampling gear.

## Technical Validation

### Geographical and water depth bias

**Fig. 2:**
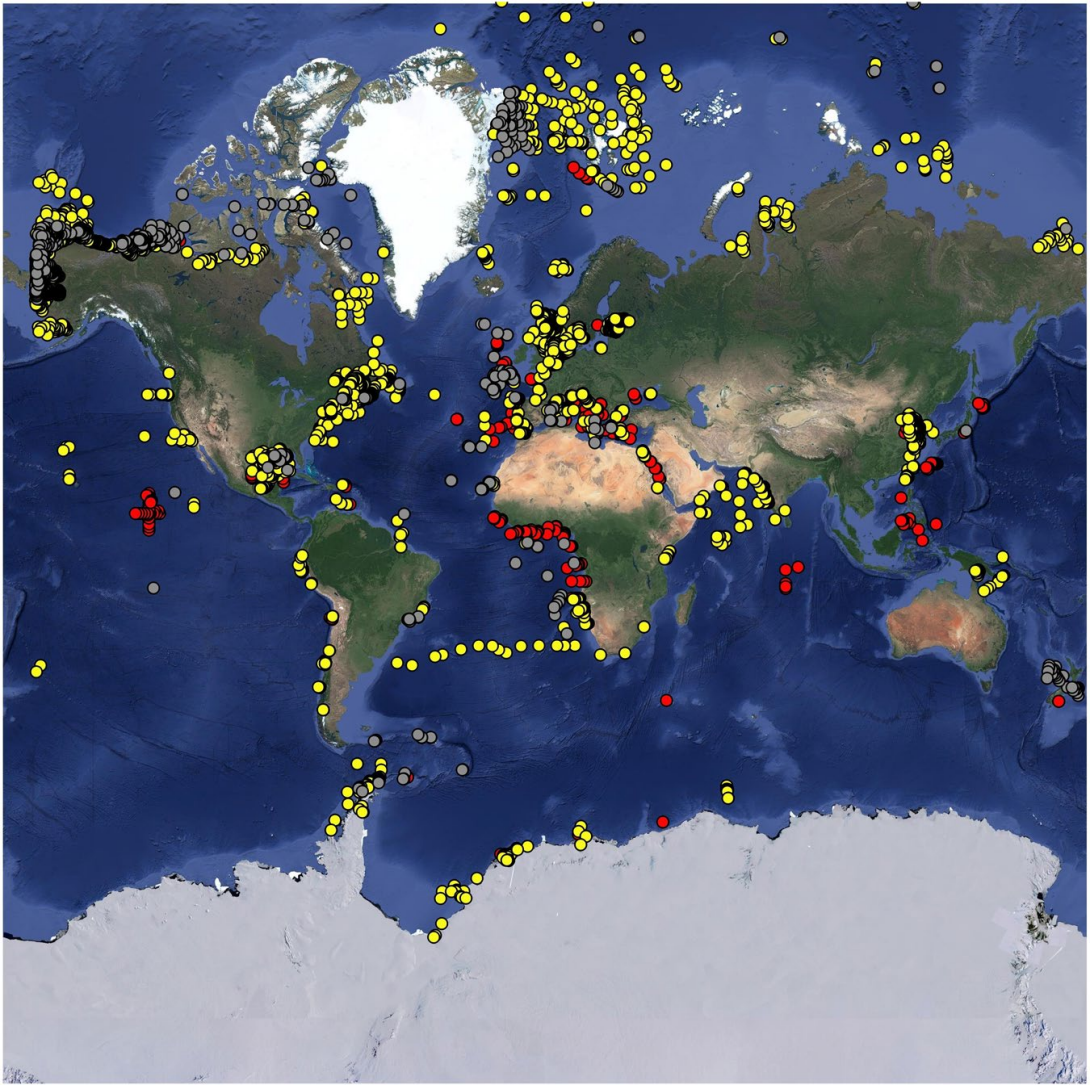
Global distribution of sampling stations where benthic biomass were sampled. Several dots represent multiple data points. Color code: red dots = metazoan meiobenthos, yellow dots = macrobenthos, grey dots = invertebrate megabenthos.

**Fig. 3:**
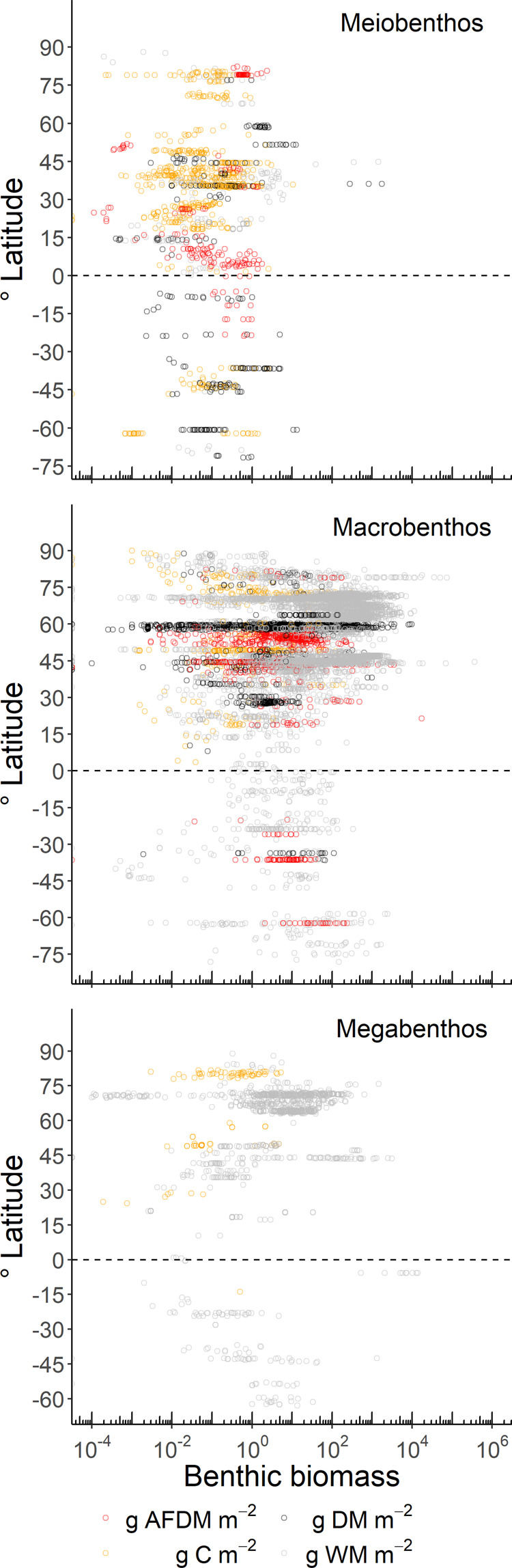
Benthic biomass (g m^−2^) of metazoan meiobenthos (upper panel), macrobenthos (middle panel), and invertebrate megabenthos (lower panel) along a latitudinal gradient. Each dot corresponds to a single biomass record and the dashed line indicates the equator. Notice the logarithmic scale on the x-axis. Abbreviations: AFDM = ash-free dry mass, DM = dry mass, C = carbon, WM = wet mass.

**Fig. 4:**
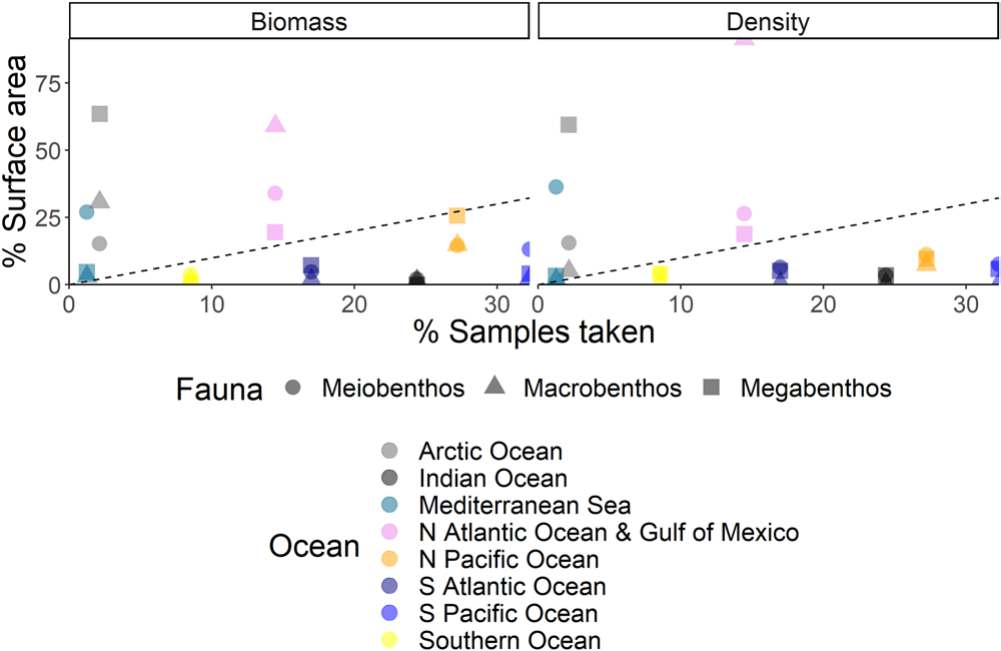
Sampling effort of meiobenthos, macrobenthos, and megabenthos  as % samples taken in relation to % surface area of the different oceans. The dashed 1:1 line indicates the equal distribution of samples over all oceans. All samples above the diagonal indicate oversampling of specific faunal biomass and/or density records, whereas all samples below the diagonal indicate undersampling.

In the database, 60% of all meiobenthic biomass records were sampled in the Atlantic Ocean (including the Gulf of Mexico and the Mediterranean Sea), 22% in the Pacific Ocean, and 12% in the Arctic Ocean (Fig. 2). Most macrobenthic samples were collected in the Atlantic Ocean (including the Gulf of Mexico and the Mediterranean Sea; 56%), with additional sampling in the Arctic Ocean (26%), and the Pacific Ocean (15%) (Fig. 2). In contrast, most megafaunal biomass data compiled in the BioBen database originated from the Arctic Ocean (50%) and the Atlantic Ocean (21%) (Fig. 2). All three benthic size classes were predominantly sampled in the northern hemisphere north of 1°N (meiobenthos: 82%, macrobenthos: 95%, megabenthos: 90%), and macrobenthos in particular was seriously undersampled south of 1°S (5% of all samples) (Figs. 3, 4). Almost no biomass samples were taken in the Indian Ocean (meiobenthos: 2%, macrobenthos: 1%, megabenthos: 0%) and the Southern Ocean (meiobenthos: 3%, macrobenthos: 1%, megabenthos: 1%) (Fig. 4). Additionally, the Pacific Ocean that represents 56% to the global ocean’s area^33^ is comparatively undersampled for macrobenthos (15% of all macrobenthos) (Fig. 4).

**Fig. 5:**
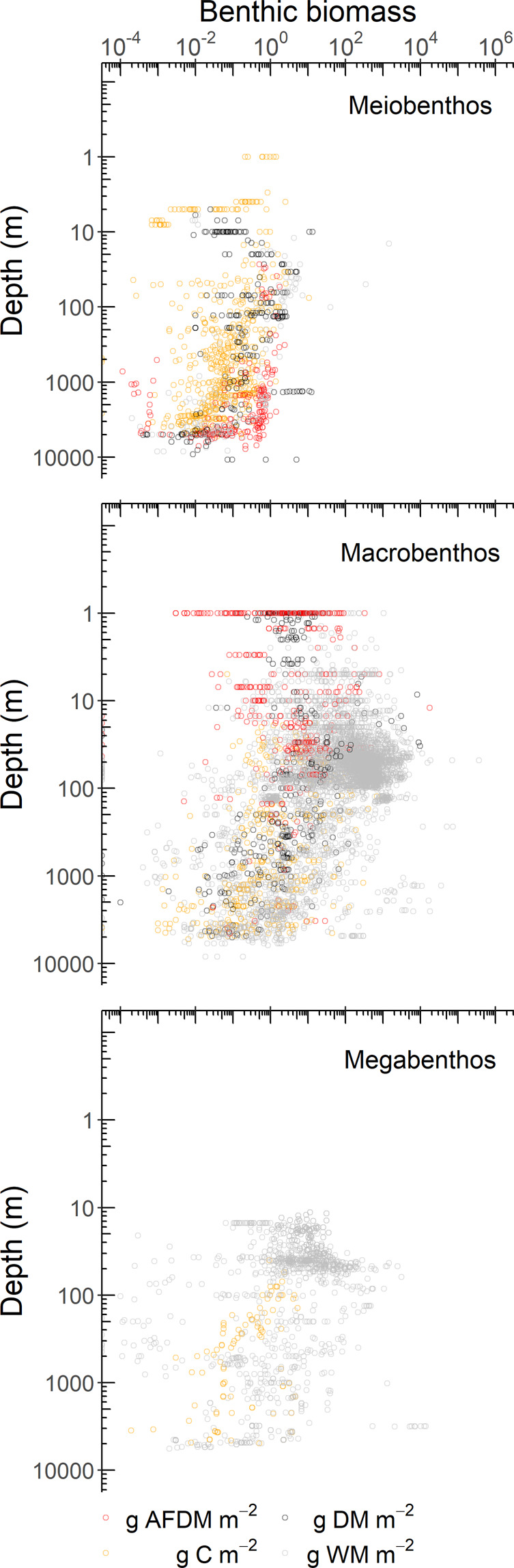
Benthic biomasses (g m^−2^) of metazoan meiobenthos (upper panel), macrobenthos (middle panel), and invertebrate megabenthos (lower panel) along a water depth gradient (m). Note the logarithmic scale on both axes. Abbreviations: AFDM = ash-free dry mass, DM = dry mass, C = carbon, WM = wet mass.

Meiobenthos biomasses were quantified mostly on the continental slope (35%) and on the continental rise *and* abyssal plains (31%) that collectively encompass 95% of the ocean seafloor^32^ (Fig. 5). In contrast, near-shore areas (29%) and continental shelves (21%; Fig. 5) dominated macrobenthic biomass samples, although these areas collectively encompass < 5% of the global seafloor^32^. In 35% of the cases no sampling depth was given in the original publications. Also 47% of all megabenthos biomass records came from areas < 50 m water depth, whereas only 10% of all megabenthos biomass samples were taken in the largest part of the seafloor, the continental rise and abyssal plains (Fig. 5). Hence, not surprisingly the benthic biomass database is biased towards shallow waters (<200 m) in the northern hemisphere, particularly, in the North Atlantic.

**Fig. 6:**
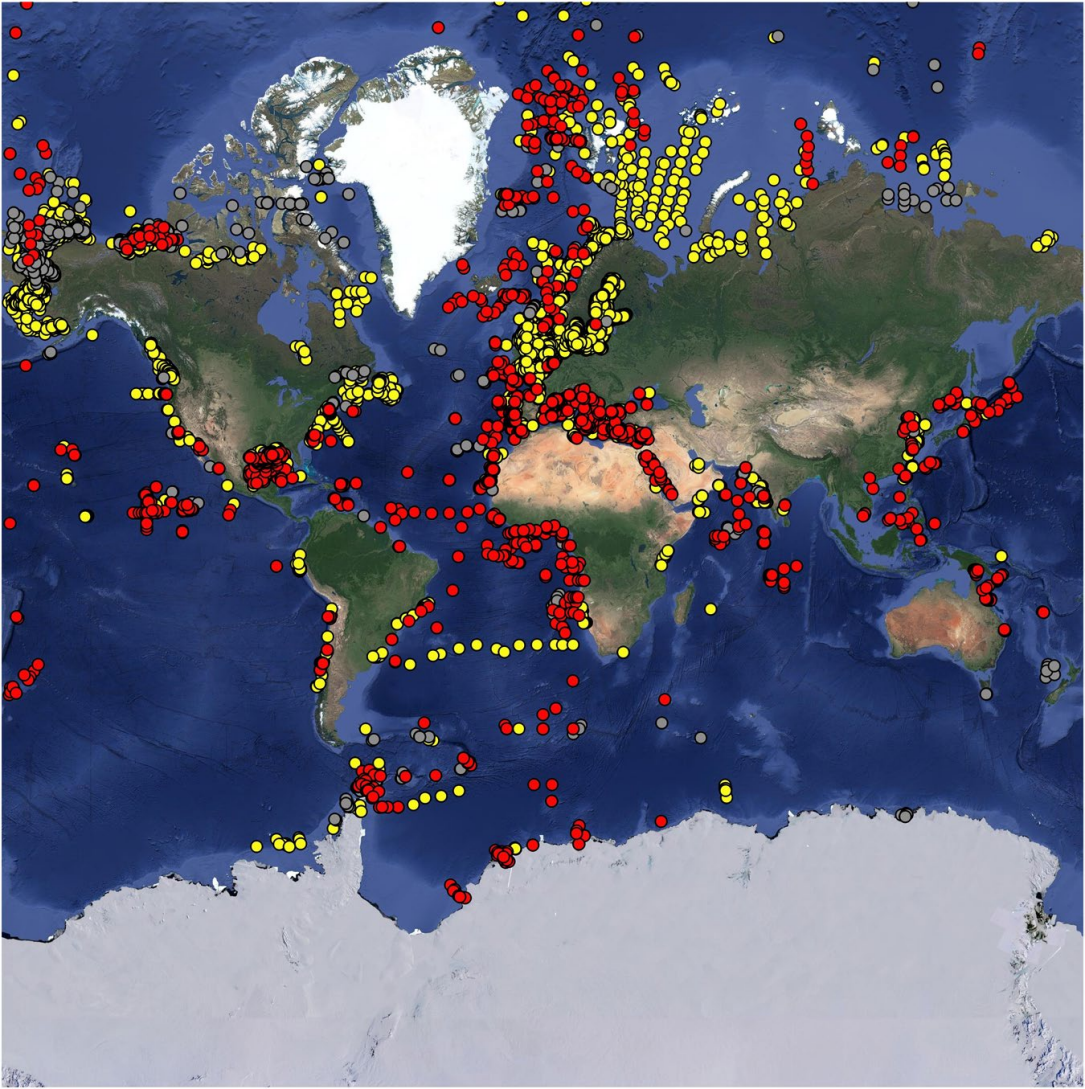
Global distribution of sampling stations where benthic densities were measured. Several dots show multiple measurements. Color code: red dots = metazoan meiobenthos, yellow dots = macrobenthos, grey dots = invertebrate megabenthos.

**Fig. 7:**
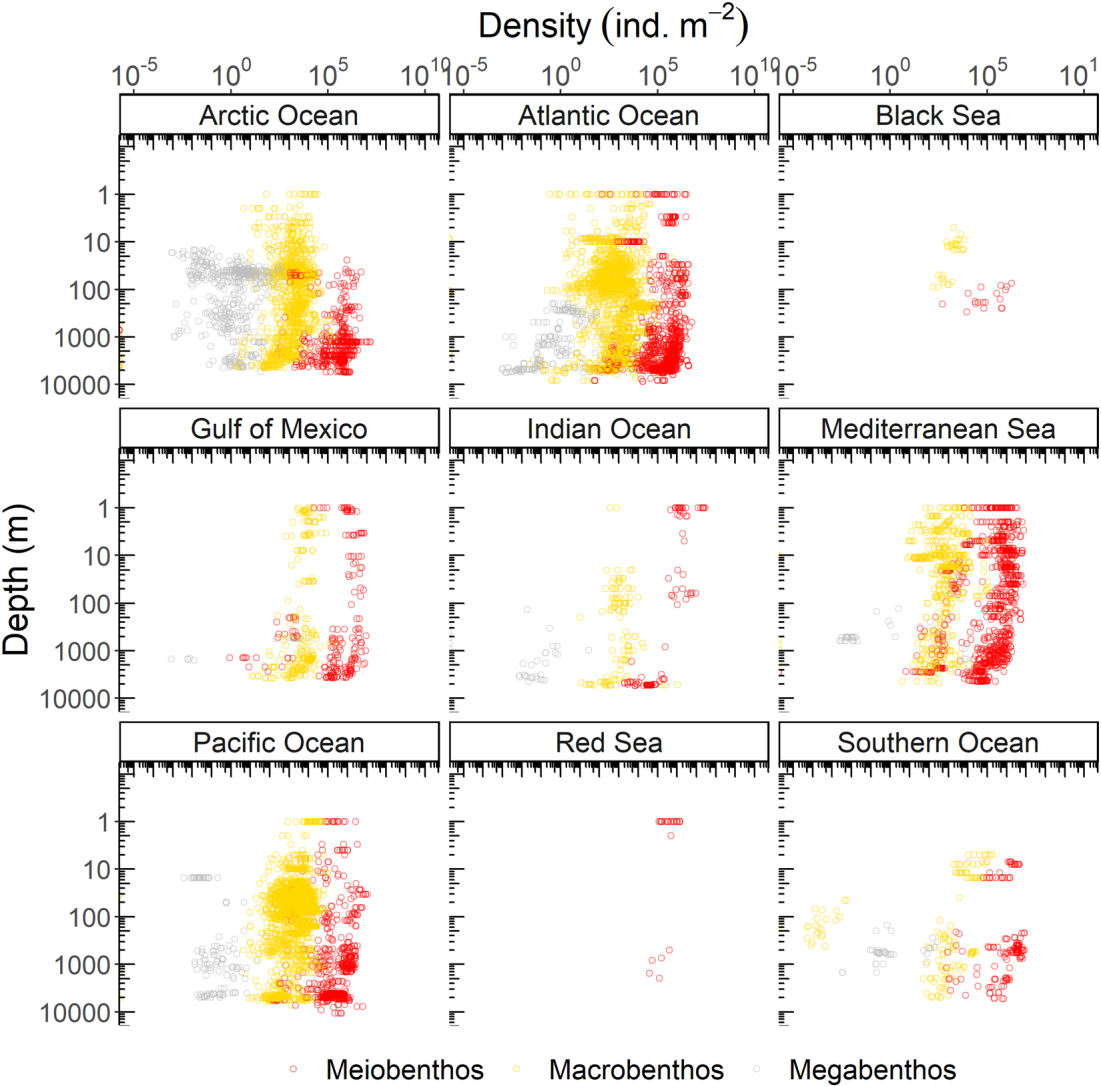
Benthic density (ind. m^−2^) of meiobenthos (red circle), macrobenthos (yellow circle), and megabenthos (grey circle) along a depth gradient (m). Note the logarithmic scale on both axes.

**Fig. 8:**
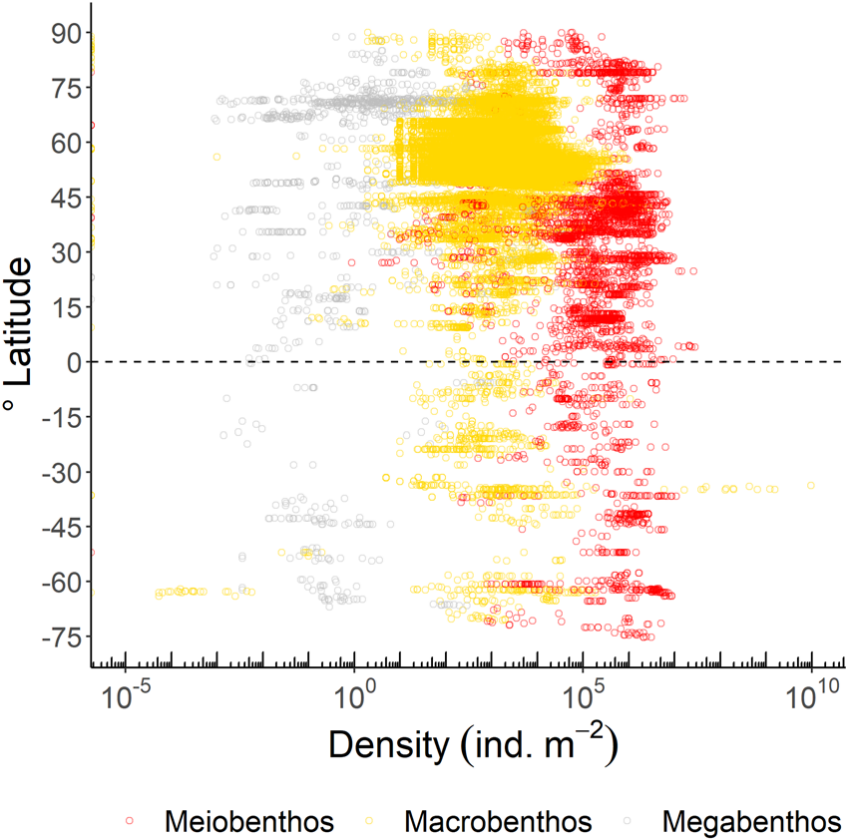
Benthic density (ind. m^−2^) of meiobenthos (red circle), macrobenthos (yellow circle), and megabenthos (grey circle) along a latitudinal gradient. Note the logarithmic scale on the x-axis.

Meiobenthic density samples were mainly taken in the Atlantic Ocean (including the Gulf of Mexico and the Mediterranean Sea; 59%) and in the Pacific Ocean (22%) (Figs. 6, 7), whereas macrobenthic density was dominantly sampled in the Atlantic Ocean (including the Gulf of Mexico and the Mediterranean Sea; 87%) (Figs. 6, 7). Megabenthic densities originated from the Arctic Ocean (53%), the Atlantic Ocean (including the Gulf of Mexico and the Mediterranean Sea; 26%), and the Pacific Ocean (14%) (Figs. 6, 7). More than 83% of all samples were taken in the northern hemisphere (>1°N), in case of macrobenthos, even 98% of all density samples were taken > 1°N (Fig. 8).

Meiobenthic and megabenthic densities were sampled to 65% and 56% at the continental slope and at the continental rise *and* abyssal plain (Fig. 7), whereas information about sampling depth was missing for 82% of the macrobenthos samples that originated predominantly from the North Atlantic. When these records are not taken into account, most of the macrobenthic density samples were collected in near-shore areas (38%) and at the continental shelf (33%). Hence, benthic density samples are biased towards the northern hemisphere and in particular towards the North Atlantic and the Arctic Ocean (Fig. 4).

### Differences in size ranges of meiobenthos, macrobenthos, and megabenthos

Metazoan meiobenthos usually includes organisms that pass through 500 μm to 1 mm mesh size and are retained on sieves with 44 μm mesh size^1^, though deep-sea biologists often use a lower mesh size limit of 32 μm for metazoan meiobenthos^34^. In our database, however, the lower mesh size limit for metazoan meiobenthos ranges from 20 μm to 74 μm, and the upper mesh size limit spans from 100 μm to 2 mm because of the different mesh sizes chosen by the authors of the original studies. Hence, some metazoan meiobenthos records include organisms that might be allocated to microbenthos, and other records that group them with macrobenthos.

Macrobenthos refers to organisms retained on a mesh of 0.5 cm, though different studies used mesh sizes between 0.5 mm and 2 mm^35^. In our database, however, authors of different studies sieved macrobenthos samples with meshes ranging from 0.25 mm to 20 mm in size. This implies, that depending on the size range used for macrobenthos, some macrobenthic records might include also be metazoan meiobenthos.

Invertebrate megabenthos are larger than macrobenthos and defined as invertebrates visible in bottom photographs (> 1 cm or > 3 cm^36^). Most megabenthic biomass and density records in the BioBenDen database lack specific information about minimum size (82% of all megabenthic biomass records and 79% of all megabenthic density records), but the studies that report a minimum size used a minimum animal length between 0.5 cm and 2 cm. Consequently, part of the megabenthic biomass and density data unavoidably might include some macrobenthos.

Therefore, researchers should consider the lower and upper sieve mesh sizes when using data from this database to ensure that the data coincide with their size requirements.

## Data Availability

The R code used to generate Figs. 3, 4, 5, 7, and 8 can be found in *Zenodo Digital Repository*^37^.

## Online-only Table

**Online-only Table 1:** Specification of the BenBioDen database with file locations.

Source	Document name	Number of studies (records)	Data description	Method
10.5061/dryad.gb5mkkwm6	List of studies for BenBio database	1,445	Alphabetical reference list of studies about metazoan meiobenthic, macrobenthic, and megabenthic biomasses that were identified following the PRISMA Statement. Additionally, it is specified which studies were excluded during the screening process and the assessment of eligibility.	Literature search
10.5061/dryad.gb5mkkwm6	List of studies for BenDen database	2,086	Alphabetical reference list of studies about metazoan meiobenthic, macrobenthic, and megabenthic densities that were identified following the PRISMA Statement. Additionally, it is reported which studies were excluded during the screening process and the assessment of eligibility.	Literature search
10.5061/dryad.gb5mkkwm6	BenBio database	384 (11,792)	All benthic biomass records compiled in the BenBio database.	Extraction of benthic biomasses records from literature
10.5061/dryad.gb5mkkwm6	BenDen database	600 (51,559)	All benthic density records included in the BenDen database.	Extraction of benthic density records from literature
